# Cost analysis of hospital-based symptomatic uterine fibroids in the kingdom of Eswatini: A prevalence-based cost-of-illness study

**DOI:** 10.1371/journal.pone.0342892

**Published:** 2026-03-04

**Authors:** Vuyisile Jabulile Ginindza, Andre Bovell, Makandwe Nyirenda, Themba G. Ginindza

**Affiliations:** 1 Discipline of Public Health Medicine, School of Nursing and Public Health, University of KwaZulu-Natal, Durban, South Africa; 2 South African Medical Research Council, Burden of Disease Research Unit, Parowvallei, Cape Town, South Africa; 3 Cancer & Infectious Diseases Epidemiology Research Unit (CIDERU), College of Health Sciences, University of KwaZulu-Natal, Durban, South Africa; Kasr Alainy Medical School, Cairo University, EGYPT

## Abstract

**Background:**

Uterine fibroids are common benign tumours of uterine smooth muscle. It has been established that uterine fibroids cause a marked global financial burden on healthcare systems and society; however, significant gaps exist in the knowledge of costs related to uterine fibroids. This study aimed to estimate costs associated with the diagnosis, care, and management of uterine fibroids in Eswatini.

**Methods:**

The study employed the Cost of Illness (COI) method, a retrospective prevalence cost model. The provider’s perspective was used to estimate all identifiable direct medical costs of UFs. Data was collected from participants’ charge and claims records from the hospitals between 21 August 2021 and 21 August 2022. The study employed a bottom-up approach to estimate all patient costs related to uterine fibroids care and treatment. The costs were calculated at the 2022 price level and converted to United States dollars (USD) using private-payer prices.

**Results:**

The total annual direct medical cost of uterine fibroids in Eswatini was estimated at USD 837894.60. This includes the total estimated costs of outpatient treatment and care, surgical treatment and care, and inpatient treatment and care. The key cost drivers were surgical and inpatient costs, USD 421583.18 and USD 110716.59, respectively.

**Conclusion:**

The total annual cost estimation of uterine fibroids care is significantly high, with the majority of the cost related to theatre and hospitalisation charges. Understanding these financial implications can help guide healthcare policies and resource management.

## Introduction

Uterine fibroids (UFs) are highly prevalent, understudied non-cancerous myometrium tumours, with a significant economic burden on women [1]. The global burden of uterine fibroids increased from 1990 to 2019, with a significant rise in incidence and disability-adjusted life years (DALYs) observed globally, whereas mortality rates associated with uterine fibroids remained relatively stable [2]. UFs are the most prevalent benign gynaecological tumours, presenting a significant healthcare burden [3]. Research findings from the sub-Saharan region revealed a significant prevalence of uterine fibroids, with 67% reported [4], while histological analysis indicated that 79% of women were affected [5]. The values observed in numerous African countries differ from the global perspective, especially regarding the prevalence values attributed to Black women. Most studies indicated UFs’ occurrences below 30% [6–8]. The common signs and symptoms are pelvic pain, menorrhagia, irregular menses, and pelvic mass and result in significant morbidity among reproductive-age women and adverse pregnancy outcomes [8,9]. There is no direct cause of UFs, but they have been associated to modifiable and non-modifiable risk factors: age, race, obesity, alcohol, caffeine, and age of menarche [10,11].

Recent studies indicated that lower socio demographic index (SDI) regions have a higher burden of UFs, while disease reduction has been noted in higher SDI regions [2]. This disparity in treatment options is particularly pronounced among low-income women, who are more likely to receive invasive treatments, leading to increased medical bills and increased emergency department visits [12,13]. Current research findings in the United States of America indicate that the economic burden of uterine fibroids has increased compared to 2010 due to new costs associated with treatment methods, Magnetic Resonance-guided Focused Ultrasound (MRgFUS) and infertility, while the majority of the burden is attributed to lost work cost [14]. Additionally, the cost of medical management has decreased directly, while surgical interventions have increased, so the financial burden of surgical treatment options for UFs continues to be extensive [14–17].

UFs’ associated costs were noticeably higher because of medication costs, surgical treatment options, and more outpatient, inpatient, and emergency room visits before and after diagnosis [18]. The economic burden of UFs in Ghana was reported to be high, related to the high prevalence, high costs linked to the surgical treatments of UFs, and the hospital stay post-surgery [19]. Though the prevalence of UFs is high in sub-Saharan Africa, there are limited research studies on UFs epidemiology, prevention, and cost [20,21]. The systematic review found that developed countries bear a significant economic burden of both direct and indirect costs of uterine fibroids, recommending further research [22]. The direct and indirect costs of UFs are enormous for both healthcare system and the individual patient, since the clients diagnosed with UFs use more healthcare resources and incur more costs during the pre- and post-diagnosis phase [23].

A study conducted in the United States of America showed that the obstetric complications of UFs contributed significantly to the increased cost of UFs treatment and care; loss of work hours mainly impacted the societal cost [24]. The type of treatment influences the cost of UFs, and hysterectomies have the most significant healthcare costs [16]. Uterine Artery embolisation (UAE) was associated with higher costs and lower quality-adjusted life years (QALYs) when compared with myomectomy in other research studies [25,26]. Kong et al. (2014) agreed with other research findings on UFs treatment cost, which identified UAE as the most effective and expensive treatment strategy, followed by the guided MRI-focused ultrasound, and then hysterectomy [27]. Hysterectomy was identified to be more costly than myomectomy because of its post-procedural complications, effects on morbidity, and quality of life [28]. The surgical management of UFs demonstrated a significant economic burden among affected individuals [15]. It is necessary to advance cost-effective treatments for the management of UFs.

Heavy menstrual bleeding (HMB) in patients with UFs was associated with considerably higher direct health care costs compared with UFs or HMB alone. In contrast, surgical and procedural costs increased diagnosis-related medical costs [29]. The cost associated with UFs also increased three times more among UFs patients with a high comorbidity index compared to those with a lower comorbidity index, as they stayed longer in the hospital [30]. Shih (2019) concurred with findings from other studies on the cost of UFs, which suggest that the cost of UFs is higher among patients with UFs [17]. A study of UFs’ cost in Ukraine discovered that UFs’ hormonal treatments were expensive [31], which contributed to the information that UFs’ treatment is costly. A study on the burden of symptomatic uterine fibroids (UFs) in Canadian women indicated that UFs had a significant financial impact [32]. Pynnä, K et al. (2021) research study noted that hospital treatment of UFs was the most expensive intervention compared to other gynaecological benign diseases [33]. In Italy, the burden and cost of UFs were reported as high; the average cost per patient was €3,249 [34].

The Eswatini Government currently assumes the primary financial responsibility for disease management, encompassing the diagnosis and treatment of UFs, while patients are required to pay only user fees. The national health policy emphasises that the Ministry of Health is responsible for defining and supporting the provision of essential healthcare packages across all service delivery levels [35]. To our knowledge, there are no recent studies on the cost of UFs in Eswatini. This study will focus on the cost estimation associated with screening, care, and treatment of UFs. The findings will provide cost information to inform sexual reproductive health (SRH) policy development on UFs’ screening, care, and treatment costs in Eswatini.

## Methods and materials

### Study setting

This study was conducted in the Kingdom of Eswatini, a sovereign state in Southern Africa, bordered by South Africa and Mozambique. The study was conducted in eight hospitals in the four geographic regions of Eswatini: Hhohho, Manzini, Lubombo, and Shiselweni. A total of eight admitting hospitals were purposely selected representative of the country’s hospitals: Piggs Peak, Mbabane Government, Mbabane Private Clinic, Raleigh Fiktin Memorial Mission, Mankayane Government, Women and Children Private, Hlatikulu Government, and Good Shepherd Catholic Mission hospitals.

### Study design

This study utilised the retrospective prevalence-based cost-of-illness to assess direct medical costs from the provider’s perspective, which is the cost covered by the government for services related to UFs. Prevalence-based cost of illness studies are identified as effective methods that assist health policy makers to develop cost containment policies. This is due to their ability to identify the primary cost components of a disease [36]. Data was collected from patient charts in the public hospitals and electronic records in the private hospitals between 21 August 2021 and 21 August 2022**.** We used the estimated UFs prevalence from our 2022 study, the prevalence and risk-associated factors of uterine fibroids in the kingdom of Eswatini, to estimate the cost of hospital-based symptomatic UFs. The analytic cross-sectional hospital-based study on the symptomatic prevalence of UFs reported a prevalence rate of 67.8% among participants. The study was conducted in eight study sites among women aged 25–64 years seeking health services at the gynaecology outpatient departments or admitted to gynaecological wards. Of the six hundred and forty-five (645) participants in the study, four hundred and thirty-seven (437) (67.8%) had an ultrasound scan confirmed UFs diagnosis. These estimations were considered the best available evidence to assess the overall burden of UFs concerning prevalent cases among the population.

### Inclusion and exclusion criteria

Medical records of women who participated in the primary UFs study and voluntarily consented, aged 25–64 years, with a confirmed UFs diagnosis, who had undergone any UFs-related medical management or surgical procedure (hysterectomy, myomectomy, laparoscopic, or uterine embolization), were included in the study. The study excluded women who were not part of the primary study, declined to participate, were under 25 or over 64 years old (due to low prevalence of UFs among this group), lacked a confirmed UFs diagnosis or UFs-related surgery or medical management.

### Management of uterine fibroids in Eswatini

The standard practice is for patients to present at the Gynaecology outpatient department presenting with signs and symptoms related to UFs, like abnormal menstruation, post-menopausal vaginal bleeding, lower abdominal pains, and pelvic heaviness. The patients are then referred to the gynaecologist, who will prescribe or perfom a pelvic ultrasound to confirm the diagnosis. If the scan indicates UFs, further management of the UFs will be determined by the patient's presenting signs and symptoms, age, desire to have children, and the size and location of the UFs in the uterus.

**Fig 1:**
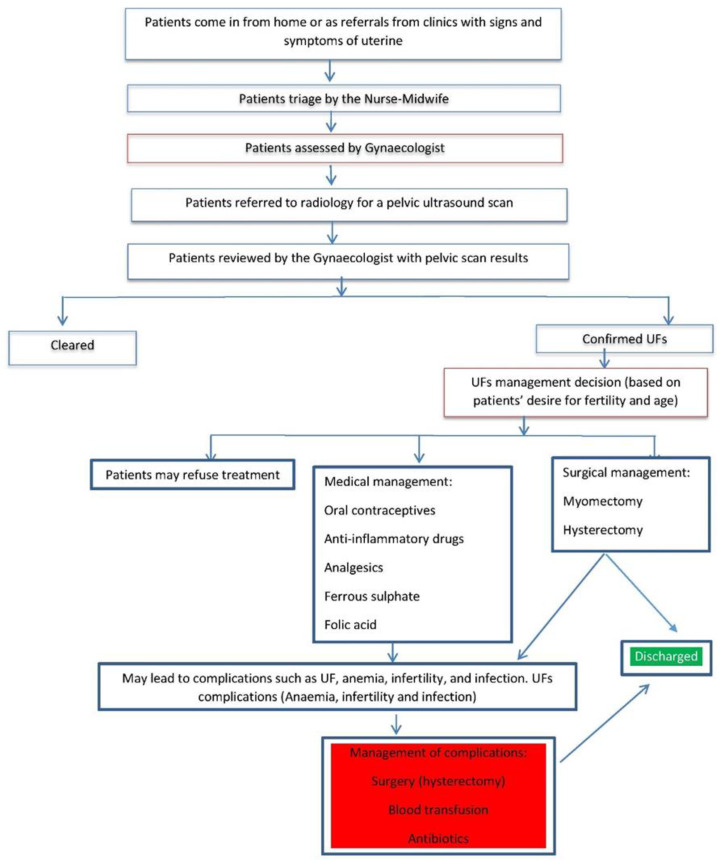
The flow diagram depicting the movement of patients diagnosed with UFs in the gynaecology department.

If the patient's age is within the childbearing range of 18–45 years, surgical removal of the uterine fibroids is done (myomectomy) to preserve fertility. However, if the patient is above 45 years and the size of the UFs is huge and in an unfavorable location, total removal of the uterus is done, open surgery (total abdominal hysterectomy). The commonly used surgical procedures in Eswatini are myomectomy and hysterectomy. Suppose the patients have reached the menopausal age, typically 50 years and above, and exhibit no threatening signs or symptoms., medical management of signs and symptoms is done without surgical intervention. UFs are also asymptomatic, so some patients are diagnosed late when they have developed complications like anaemia and infertility. The anaemic patients are transfused blood before any surgical intervention, and infertility management varies from patient to patient. The current flow of patients diagnosed with UFs in the gynaecology department is shown as (Fig 1), in the supplementary files folder.

Furthermore, recent research findings on experiences of patients diagnosed with uterine fibroids in Eswatini revealed their beliefs and perceptions, such as the belief in traditional medicine to cure the disease. Additionally, lack of knowledge about UFs, dissatisfaction with the current treatment options, and fear of complications negatively influenced the health-seeking behaviour [37]. These contributed to delay in treatment and complications of UFs.

### Data collection

Data on UFs’ cost was accessed from the participants files. Data was collected from participants’ charge and claim sheets in the hospitals through record extraction and electronic records. Consistent with the study design [36], the principal researcher accessed the patients’ records in the different study sites between 21 August 2021 and 21 August 2022. We used files of patients who had consented to partake in the primary research: the prevalence and risk-associated factors of uterine fibroids in Eswatini. The UFs cost estimation data was collected from the four hundred and thirty-seven (437) (67.8%) confirmed UFs participants’ health records. Table 1 summarises the sources of data and prices.

**Table 1:** Source of cost data.

Parameter	Care Components	Source of cost	Price source
Outpatient	Consultation	Patients file	Private hospital
	Ultrasound scan & Diagnosis	Patients file	Private hospital
	Drug Treatment	Patients file	Private hospital
Inpatient	Hospitalization	Patients file	Private hospital
	Blood transfusion	Patients file	Private hospital
	General Medical Supplies	Patients file	Private hospital
	Drugs	Patients file	Private hospital
Surgical	Theatre gases	Patients file	Private hospital
	Anaesthesia	Patients file	Private hospital
	Drugs	Patients file	Private hospital
	Wound Care	Patients file	Private hospital

### Method of costing

The study employed a cost-of-illness methodology using a prevalence-based cost model, incorporating the bottom-up approach to estimate the direct medical costs associated with uterine fibroids [38]. The bottom-up approach estimated the cost associated with outpatient, inpatient, and surgical care based on the costs of individual units of service performed. All identifiable direct medical costs were considered for UFs. To estimate the unit cost of each service rendered, this formula was used; this included two subcategories: (a) direct medical costs, which included tests such as complete blood count, medications, surgery, medical supplies, and emergency services, and (b) direct non-medical costs, including hospitalisation costs and meal costs while admitted. Direct medical costs were calculated based on outpatient visits, laboratory tests, radiographic tests, medications, and surgery. The cost of each care element was multiplied by the total number of patients identified within that field. All cost-generating events were identified and ascribed to monetary value based on private pricing from the private sector hospitals. The following formulas were used to estimate the cost of UFs:


Total Direct Medical Cost=Σ (Unit Cost of Resource×Quantity of Resource Used) 

Total Direct Non−Medical Costs=∑ (Cost of hospitalization+meals)

Total cost of UFs: Total Estimated Cost of UFs=Σ (Cost of each component).

All costs were computed at the 2022 price level and converted to US dollars ($). We used private payer pricing since the public had no available unit pricing system in place. The average SZL/USD exchange rate for 2022 of 0.0614 US dollars per Swaziland Lilangeni (SZL) was used [39].

Formula:  Direct Medical Cost=(Cost of specific medical services x Quantity of services)

Aside from presenting estimates in USD, estimates were also presented in the local currency so as to showcase the original values of actual transactions prior to translation gains or losses.

### Sensitivity analysis

A sensitivity analysis was conducted utilizing a ± 25% variation to address uncertainties in cost estimations. The analysis was conducted following the conclusion of the UFs cost estimation to assess the validity and robustness of the initial findings. This was accomplished by determining whether the results remained consistent when alternative assumptions regarding data or methods were employed [40,41]. The sensitivity analysis yielded similar findings to the initial findings.

### Ethical consideration

Ethical approval was obtained from the University of KwaZulu-Natal Biomedical Research Ethics Committee (BREC), BREC/00002571/2021, and the Eswatini Health and Human Research Review Board (EHHRRB), EHHRRB023/2021. Permission was obtained from the proposed research sites (gatekeepers), regional health administrators, chief executive officers, matrons, and nurse managers. Participation in the research study was voluntary. Individuals selected for the study were taken through an informed consent process, including information on the research purpose. All participants individually consented verbally and signed a written consent form. Participants were assigned pseudonyms to ensure confidentiality*.*

## Results

### The diagnosis profile of the participants’ health records

Among the four hundred and thirty-seven (437) (67.8%) participants’ records confirmed with UFs, two hundred and eighty-six patients (286) (65.4%) were treated as outpatients, and one hundred and fifty-one (151) (30.5%) were admitted as inpatients (Table 2). Of the one hundred and fifty-one (151) inpatients, eighteen [18] (4.1%) had anaemia related to UFs, and one hundred and thirty-three (133) (30.4%) underwent myomectomy or hysterectomy, as shown in Table 2.

**Table 2:** The diagnosis profile of the participants’ health records.

Diagnosis profile	Total health records	UFs as % of Total (67.8)
**UFs**	437	67.8
**Inpatient**	151	30.5
**UFs complication (Anemia)**	18	4.1
**Surgery**	133	30.4
**Outpatient**	286	44.3

### Estimated direct medical costs of UF care and treatment costs

Table 3 summarises the estimated outpatient UFs care and treatment costs. The estimated direct medical outpatient cost was USD 110,716.34 (range 83,037.44 and 138,395.74). Based on the care parameters identified, the leading cost drivers were pelvic ultrasound scans, at USD 48,297.24 (range: USD 36,222.93 to 60,371.55), and Gynaecologist consultations, at USD 42,260.09 (range: USD 31,695.06 to 52,825.11). Treatment costs linked to outpatient care were USD 7,850.98 (range: USD 5,888.24 and 9,813.73). The total outpatient average costs of all care components were calculated in USD using this formula:

**Table 3:** The estimated outpatient UFs care and treatment costs.

	Activities	Prevalence (2021–2022)	Quantity	Unit cost (SZL)	Total cost (SZL)	Unit cost (US$)	Total cost (US$)	Range of Total	
								Cost (US$) +/- 25%	
								Lower (−25%)	Upper (+25%)
**Consultation**	Gynecologist’s fee	437	3	525.00	688,275.00	32	42,260.09	31,695.06	52,825.11
**Imaging and diagnosis**	Ultasound scan	437	1	1,800.00	786,600.00	111	48,297.24	36,222.93	60,371.55
**Laboratory tests**	Full blood Count	437	3	148.00	194,028.00	9	11,913.32	8,934.99	14,891.65
	Blood glucose test strips	437	1	14.72	6,432.64	1	394.96	296.22	493.71
	**Sub-total**			**162.72**	**200,460.64**	**10**	**12,308.28**	**9,231.21**	**15,385.35**
**Drugs and treatment**	Paracetamol 5oomg tablets	437	40	0.19	3,321.20	0	203.92	152.94	254.90
	Ferrous sulphate	437	30	3.00	39,330.00	0	2,414.86	1,811.15	3,018.58
	Folic acid	437	30	3.00	39,330.00	0	2,414.86	1,811.15	3,018.58
	Brufen 400 mg	437	15	3.00	19,665.00	0	1,207.43	905.57	1,509.29
	Ovral	437	30	2.00	26,220.00	0	1,609.91	1,207.43	2,012.39
	Sub-total			11.19	127,866.20	1	7,850.98	5,888.24	9,813.73
	**Total**			**2,498.91**	**1,803,201.84**	**153**	**110,716.59**	**83,037.44**	**138,395.74**

**Quantity—represents the number of services per year.**


$$\begin{array}{c} \;average\;=\left(\frac{consultation+ultrasound\;scan+laboratory+drugs}{N}\right)=\frac{\;\text{1}\text{1}\text{0},\text{7}\text{1}\text{6}.\text{3}\text{4}\;}{\text{4}\text{3}\text{7}}=\$\text{253}.\text{36} \end{array}$$

**Fig 2:**
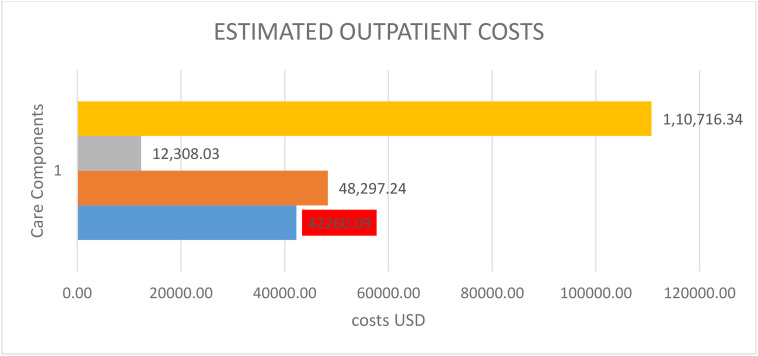
The Summary of outpatient costs (US$).

Table 3 and (Fig 2) show the estimated outpatient UFs care and treatment costs.

### Estimated direct medical costs of UFs’ surgical care and treatment costs

**Fig 3:**
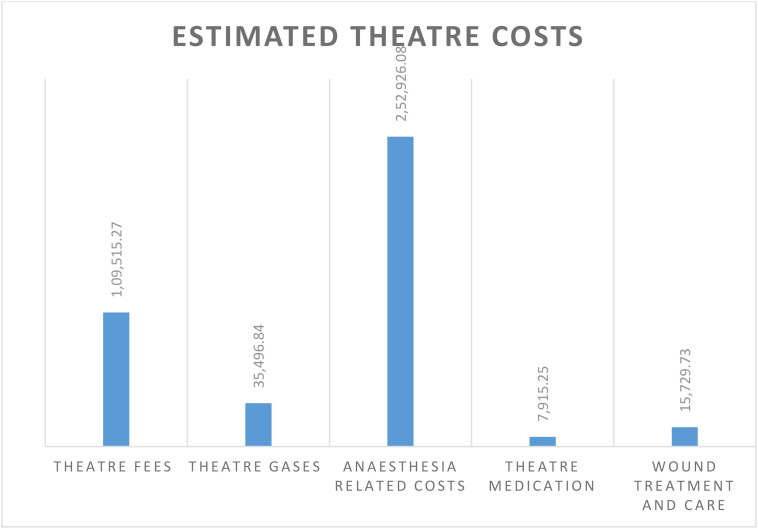
The Summary of theatre costs.

Table 4 summarises the estimated costs of surgical treatment and care for UFS. This was the primary annual cost driver among all care parameters, at USD 421,583.18 (range: USD 316,187.38 to 526,978.97). One hundred and thirty-three (133) participants underwent either myomectomy or hysterectomy to remove the UFs. The main cost drivers were anesthesia-related expenses at USD 252,926.08 (range: USD 189,694.56 to 316,157.60) and theatre fees at USD 109,443.93 (range: USD 82,082.95 to 136,804.91), as shown in Table 4 and (Fig 3).

**Table 4:** The estimated costs of UFs’ surgical care and treatment.

	Activities	Prevalence (2021–2022)	Quantity	Unit cost (SZL)	Total cost (SZL)	Unit cost (US$)	Total cost (US$)	Range of Total	
								Cost (US$) +/- 25%	
								Lower (−25%)	Upper (+25%)
**Theatre fees**	Theatre fees	**133**	**130**	**103.16**	**1,783,636.40**	**6.33**	**109,515.27**	82,136.46	136,894.09
**Theatre gases:**	Oxygen &nitrous oxide	133	127	8.21	138,675.11	**0.50**	8,514.65	6,385.99	10,643.31
	Ultane	133	127	24.98	421,937.18	**1.53**	25,906.94	19,430.21	32,383.68
	Oxygen in recovery	133	1	26.37	3,507.21	**1.62**	215.34	161.51	269.18
	Oxygen in theatre	133	130	0.81	14,004.90	**0.05**	859.90	644.93	1,074.88
	**Sub-total**			**60.37**	**578,124.40**	**3.71**	**35,496.84**	26,622.63	44,371.05
**Anaesthesia related costs:**	Esmeron 50 mg inj	133	1	175.35	23,321.55	10.77	1,431.94	1,073.96	1,789.93
	Genius 2SGLuse probe cover	133	4	1.38	734.16	0.08	45.08	33.81	56.35
	Propofol% in 20mils	133	5	6,062	4,031,230.00	372.21	247,517.52	185,638.14	309,396.90
	Propofol 1% injection	133	1	30.31	4,031.23	1.86	247.52	185.64	309.40
	Robinul 0.2 mg	133	1	21.31	2,834.23	1.31	174.02	130.52	217.53
	Filter guard with luer lock	133	1	27.59	3,669.47	1.69	225.31	168.98	281.63
	Airway size 4	133	1	23.06	3,066.98	1.42	188.31	141.23	235.39
	Endotracheal tubes 7 mm cuffed	133	1	120.49	16,025.17	7.40	983.95	737.96	1,229.93
	Electrode ECG foam	133	3	2.56	1,021.44	0.16	62.72	47.04	78.40
	Electro- surgical pad adult Rem covidien	133	1	226.77	30,160.41	13.92	1,851.85	1,388.89	2,314.81
	Suction catheter	133	1	24.23	3,222.59	1.49	197.87	148.40	247.33
	**Sub-total**			**6715.05**	**4,119,317.23**	**412.30**	**252,926.08**	189,694.56	316,157.60
**Theatre medication**	Tramadol100mg/2mil	133	1	37.52	4,990.16	**2.30**	306.40	229.80	382.99
	Cefetrizole 1g	133	2	24.93	6,631.38	**1.53**	407.17	305.38	508.96
	Lignocaine 2% in 20mil	133	5	1.12	744.80	**0.07**	45.73	34.30	57.16
	Remicaine (lignocaine12% inj 5mil)	133	1	8.12	1,079.96	**0.50**	66.31	49.73	82.89
	Adrenalin 1 mg	133	1	5.22	694.26	**0.32**	42.63	31.97	53.28
	Morphine 10 mg/ml in	133	1	9.36	1,244.88	**0.57**	76.44	57.33	95.54
	Diclofenac 100 mg suppository	133	1	6.39	849.87	**0.39**	52.18	39.14	65.23
	Neostigmine 2.5 mg injection	133	1	14.6	1,941.80	**0.90**	119.23	89.42	149.03
	Labetalol 5 mg	133	2	416.3	110,735.80	**25.56**	6,799.18	5,099.38	8,498.97
	**Subtotal**			**523.56**	**128,912.91**	**32.15**	**7,915.25**	5,936.44	9,894.07
**Wound treatment and care**	U/S cutting and coagulation device	133	1	444.65	59,138.45	**27.30**	3,631.10	2,723.33	4,538.88
	Abdominal swabs x-ray 450x370	133	5	49.176	32,702.04	**3.02**	2,007.91	1,505.93	2,509.88
	Diathermy pencil reliant	133	1	152.49	20,281.17	**9.36**	1,245.26	933.95	1,556.58
	Administration set adult 20drp	133	1	22	2,926.00	**1.35**	179.66	134.74	224.57
	Opsite iv 3000	133	1	23.47	3,121.51	**1.44**	191.66	143.75	239.58
	Iv cannular 20 port and wing	133	1	8.66	1,151.78	**0.53**	70.72	53.04	88.40
	Syringe 5mils	133	3	1.78	710.22	**0.11**	43.61	32.71	54.51
	Syringe 10 mils	133	1	1.91	254.03	**0.12**	15.60	11.70	19.50
	Syringe 20mils	133	2	3.92	1,042.72	**0.24**	64.02	48.02	80.03
	Syringe 3 mils	133	3	1.39	554.61	**0.09**	34.05	25.54	42.57
	Needles 18 G	133	4	0.57	303.24	**0.03**	18.62	13.96	23.27
	Needles 21 G	133	4	0.53	281.96	**0.03**	17.31	12.98	21.64
	Needles 23 G	133	2	0.5	133.00	**0.03**	8.17	6.12	10.21
	Gauze swabs 100x100	133	1	20	2,660.00	**1.23**	163.32	122.49	204.16
	Clinical lubricating jelly 2.5 g sachets	133	1	1.36	180.88	**0.08**	11.11	8.33	13.88
	Sodium chloride 1000mls	133	1	27.88	3,708.04	**1.71**	227.67	170.76	284.59
	Ringer’s lactate 1000mls	133	1	37.35	4,967.55	**2.29**	305.01	228.76	381.26
	Water for injection	133	2	4.55	1,210.30	**0.28**	74.31	55.73	92.89
	Gloves sterile comfit 7.5	133	2	12.075	3,211.95	**0.74**	197.21	147.91	246.52
	Gloves Biogel size 7.5	133	2	13.94	3,708.04	**0.86**	227.67	170.76	284.59
	Scalpel blade	133	2	0.615	163.59	**0.04**	10.04	7.53	12.56
	Suction catheter	133	1	24.23	3,222.59	**1.49**	197.87	148.40	247.33
	Foley catheter FG 22	133	1	28.28	3,761.24	**1.74**	230.94	173.21	288.68
	Receptal liner blue	133	1	137.5	18,287.50	**8.44**	1,122.85	842.14	1,403.57
	Razor disposable	133	1	5.5	731.50	**0.34**	44.91	33.69	56.14
	Imagine dressing 9.5 cm x9.5cm	133	1	74.65	9,928.45	**4.58**	609.61	457.21	762.01
	Primapore dress 2000mmx100mm	133	1	19.53	2,597.49	**1.20**	159.49	119.61	199.36
	301−32 Synthabs tap 90 cm x40mm	133	4	75.52	40,176.64	**4.64**	2,466.85	1,850.13	3,083.56
	301−23 suture vicryl	133	1	75.52	10,044.16	**4.64**	616.71	462.53	770.89
	320−23 suture Synthabs PGA Violet 2/0, 90 cm 1/2	133	2	75.52	20,088.32	**4.64**	1,233.42	925.07	1,541.78
	Dressing pack	133	1	33.75	4,488.75	**2.07**	275.61	206.71	344.51
	Urine bag	133	1	3.36	446.88	**0.21**	27.44	20.58	34.30
	**Subtotal**			**1382.176**	**256,184.60**	**84.87**	**15,729.73**	11,797.30	19,662.17
**TOTAL**	**TOTAL**			**8,784.32**	**6,866,175.54**	**539.36**	**421,583.18**	316,187.38	526,978.97

### Estimated direct medical costs of UFs inpatient and treatment care

**Fig 4:**
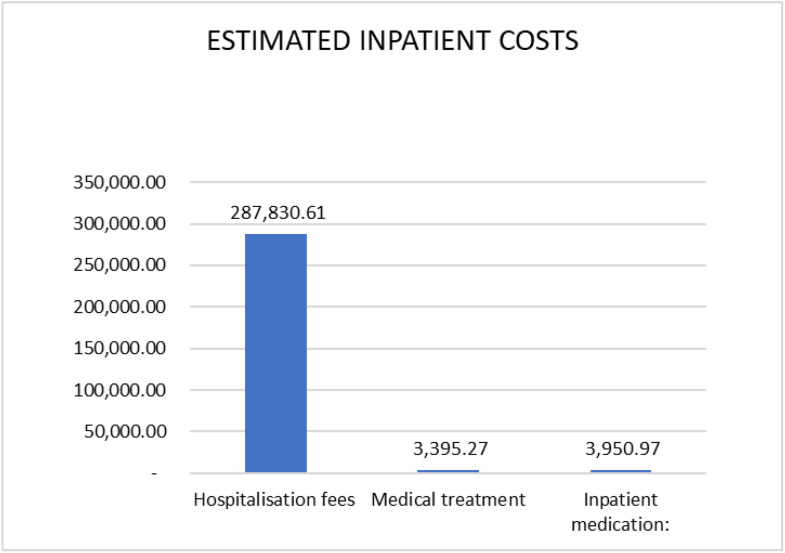
The summary of inpatient costs.

Table 5 provides a summary of UFs’ annual estimated inpatient costs. Among the participants confirmed with UFs, one hundred fifty-one (151) were treated as inpatients. They were admitted for an average of 3.5 days. The total estimated UFs’ inpatient treatment and care cost was USD 305,54.89 (range USD 229,196.17 and 381,993.62). The leading cost driver was hospitalisation fees, at USD 287,830.61 (range: USD 215,872.96 to 359,788.27), shown in Table 5, and (Fig 4) in the supplementary files folder.

**Table 5:** The estimated cost of UFs inpatient and treatment, and care.

	Activities	Prevalence (2021–2022)	Quantity	Unit cost (SZL)	Total cost (SZL)	Unit cost (US$)	Total cost (US$)	Range of Total	
								Cost (US$) +/- 25%	
								Lower (−25%)	Upper (+25%)
**Hospitalisation fees**	**hospitalisation fees**	151	**3.5**	**8,870**	**4,687,795.00**	544.62	**287,830.61**	215,872.96	359,788.27
**medical treatment:**	Genius 2SGL use probe cover	151	5	1.38	1,041.90	0.08	63.97	47.98	79.97
	Accugence blood glucose test strips	151	4	14.72	8,890.88	0.90	545.90	409.43	682.38
	Water for injection	151	1	4.55	687.05	0.28	42.18	31.64	52.73
	Ringer’s lactate 1000mils	151	2	37.35	11,279.70	2.29	692.57	519.43	865.72
	Clinical lubricating jelly 2,5g sachet	151	1	1.36	205.36	0.08	12.61	9.46	15.76
	Adult administration set 20drp	151	1	22	3,322.00	1.35	203.97	152.98	254.96
	Dressing pack	151	1	33.75	5,096.25	2.07	312.91	234.68	391.14
	Linen saver	151	1	3.36	507.36	0.21	31.15	23.36	38.94
	Foleys catheter FG 12, silicon coated	151	1	36.25	5,473.75	2.23	336.09	252.07	420.11
	Iv cannula 20 G port and wing	151	1	8.66	1,307.66	0.53	80.29	60.22	100.36
	I v cannula 22G	151	1	10.29	1,553.79	0.63	95.40	71.55	119.25
	Needles 18G	151	3	0.57	258.21	0.03	15.85	11.89	19.82
	Needles 21 G	151	3	0.53	240.09	0.03	14.74	11.06	18.43
	Syringe 5mils	151	3	1.78	806.34	0.11	49.51	37.13	61.89
	Syringe 10mls	151	3	1.91	865.23	0.12	53.13	39.84	66.41
	Opsite IV 3000	151	1	23.47	3,543.97	1.44	217.60	163.20	272.00
	Syringe 5mils	151	3	1.78	806.34	0.11	49.51	37.13	61.89
	Syringe 10mls	151	3	1.91	865.23	0.12	53.13	39.84	66.41
	Adult urine bags	151	1	25	3,775.00	1.54	231.79	173.84	289.73
	Sodium chloride 50 mils	151	3	15.6	7,066.80	0.96	433.90	325.43	542.38
	**Subtotal**			**246.22**	**57,592.91**	15.12	**3,536.20**	2,652.15	4,420.26
**Transfusion**	crossmatch	151	1	118	17,818.00	7.25	1,094.03	820.52	1,367.53
	Blood transfusion	151	2	466.4	140,852.80	28.64	8,648.36	6,486.27	10,810.45
	**Sub-total**			**584.4**	**158,670.80**	35.88	**9,742.39**	7,306.79	12,177.98
**Inpatient medication:**	Metoclopramide 10 mg injection	151	3	8.12	3,678.36	0.50	225.85	169.39	282.31
	Ondansetron 4 mg	151	10	23.53	35,530.30	1.44	2,181.56	1,636.17	2,726.95
	Paracetamol 10 mg infusion	151	3	32.94	14,921.82	2.02	916.20	687.15	1,145.25
	Tramadol 100 mg injection	151	3	38	16,996.56	2.30	1,043.59	782.69	1,304.49
	Diclofenac 100 mg suppository	151	2	6.39	1,929.78	0.39	118.49	88.87	148.11
	Amoxiclav tablets	151	10	18.32	27,663.20	1.12	1,698.52	1,273.89	2,123.15
	**Subtotal**			**108.5**	**73,056.82**	6.66	4,485.69	3,364.27	5,607.11
	**TOTAL**			**9,224.72**	**4,977,115.53**	**566.40**	**305,594.89**	229,196.17	381,993.62

### Overall UFs’ estimated cost

The total annual estimated UFs’ cost was USD 837,894.66 (between USD 628,421.00 and USD 1,047,368.33). The leading contributor to the annual direct medical costs was surgical fees at USD 421,583.18 (ranging between USD 316,187.38 and USD 526,978.97), followed by inpatient fees at USD 305,594.89 (ranging between USD 229,196.17 and USD 381,993.62), and the outpatient fees at USD 110,716.59 (ranging between USD 83,037.44 and USD 138,395.74), shown in Table 6.

**Table 6:** The overall UF estimated cost.

Activities	N	Unit cost (SZL)	Total cost (SZL)	Unit cost (US$)	Total cost (US$)	Total average costs (US$)	Total Cost %	Range of Total	
								Cost (US$) +/- 25%	
								Lower(−25%)	Upper (+25%)
**Surgical costs**	133	8,784.32	6,866,175.54	539.36	421,583.18	3,169.80	0.50	316,187.38	526,978.97
**Inpatients costs**	151	9,224.72	4,977,115.53	566.40	305,594.89	2,023.81	0.36	229,196.17	381,993.62
**Outpatients costs**	437	2,498.91	1,803,201.84	153.43	110,716.59	253.36	0.13	83,037.44	138,395.74
**TOTAL**		20,507.95	13,646,492.91	1,259.19	837,894.66	5,446.96		628,421.00	1,047,368.33

## Discussion

### Key results

This study presents an analysis of the direct medical costs of hospital-based symptomatic UFs in the kingdom of Eswatini. The results suggest that the overall annual direct medical costs of UFs were USD 837,894.66 in 2022. The leading cost driver was theatre treatment and care, which accounted for almost 50% of the total cost (USD 421,583.18). This was followed by hospitalisation and outpatient costs at 36% (USD 305,594.89) and 13% (USD 110,716.59), respectively. The findings from this study reveal that the estimates of the annual direct medical costs are considerably high, considering that the government is responsible for most of the health costs and the total health expenditure has been increasing [42]. In Eswatini, the government finances approximately 45% of the total health expenditure, whereas out-of-pocket (OOP) contributions by individuals constitute a modest 10%. This low OOP percentage suggests that the majority of the direct cost burden does not come from individual citizens at the point of care [43]. Moreover, the financial health protection in Eswatini is insufficient, despite the fact that out-of-pocket payments account for a minor percentage (<15.0%) of the country’s total health finance [42]. Additionally, Eswatini has been identified as one of the African countries vulnerable to external shocks that can impact health financing, the performance of the health system, and health outcomes [44]. The methodology used also contributed to the high cost; the private costing, so we concur with the research findings from Eswatini that emphasized that the country needs to consider financing options that assure fair access to needed quality health care without causing undue hardship on the poor and vulnerable [42].

### Interpretations and existing literature

Findings from previous but similar research studies have reported a high direct cost of UFs [17,18,34]. Although the cost estimates for UFs in this study are high, our results are 5–7 times lower than those reported in America and Italy [22,34,45]. This observed difference may have been influenced by the socio-economic status of the referenced countries and variations in the research methodologies employed. Our study focused entirely on direct medical costs, whereas the others included both direct and indirect costs. Though the prevalence of UFs is high in the sub-Saharan region, there are notably fewer similar studies on the cost estimation of UFs. A research study in Ghana reported that due to the high prevalence of UFs, and the increased number of UFs-related surgeries, the medical cost of UFs is likely to be high [19]. This emphasizes the importance of conducting additional research on the cost of UFs treatment in Eswatini.

Our results showed that the highest cost emanated from the UFs surgical treatment and care, with the common surgical management being hysterectomy and myomectomy. These findings are similar to those of previous UFs research findings that reported the surgical management of UFs as a significant financial burden [15,16,46,47]. Further research to explore alternative cost-effective treatments for UFs is recommended.

The other high costs emanated from inpatient treatment and care, USD 293,064.30. Most patients were hospitalised after the surgical intervention of UFs or due to the direct complications of UFs. The main complication was anaemia; patients were admitted for blood transfusions. We concur with the findings from previous studies, which observed that UFs’ complications increase the economic burden of the disease [24,29,45]. Early identification and treatment of UFs will prevent complications, thus reducing the unnecessary increase in UFs’ cost. Advocating for the introduction of early UFs screening for all women of reproductive age through Sexual Reproductive Health (SRH) policymakers will improve the management and care of UFs.

The UFs’ outpatient cost was the lowest, USD 110,716.59. The outpatient treatment and care costs remained low despite the large number of participants treated at the outpatient centres. This finding is similar to those from other studies, which have observed that outpatient or medical UFs treatments have a lower financial burden compared to invasive or surgical procedures [14,16,17,48]. We agree with the findings from a study that highlighted that the evasive procedures should be minimised, reserved for patients with heavy symptomatology, and introduce new UFs therapeutic options to minimise cost.

In Eswatini, there are no prescribed treatment guidelines for UFs, and most patients prefer the non-surgical management of UFs. We recommend future research in the country that will look into the new UFs treatment approaches, such as patient-centred care, treatment guidelines which and pharmacological and surgical management alternatives. This will lessen the likelihood of haphazard or delayed treatments, which could raise the disease’s financial impact.

Considering the high prevalence of UFs, we had hypothesised that the cost of UFs treatment and care would be higher than other reproductive health conditions among women in Eswatini. Contrary to that, other research findings have revealed a much higher cost of cervical cancer and papillomavirus [49,50]. Though our findings suggest that the annual estimated cost of UFs in the country is less than the cost of other reproductive system diseases, UFs cost has a substantial economic impact on patients, health systems, and the government of the Kingdom of Eswatini. New longitudinal research is recommended that will cover both the direct and indirect cost estimates of UFs.

## Implications

The findings of this study will be used to enhance awareness among the SRH policymakers regarding the economic burden posed by UFs and identify policy strategies to alleviate the burden. The introduction of updated clinical practice guidelines, with evidence-based medical and surgical treatment options, will significantly enhance the management of fibroids. Furthermore, investing in alternative, cost-effective treatments for UFs in the country is crucial to mitigate the financial and physical burden of the disease. More importantly, the implementation of index costing in public hospitals will improve future cost estimates of disease.

## Strengths

There were several notable strengths associated with this work. Firstly, this is the first study to estimate the costs of UFs in the country. This study aims to create awareness among SRH policymakers and influence the development of cost-effective UFs treatment strategies. Furthermore, based on patient files, all diagnoses of UFs were confirmed by an ultrasound scan, thereby further validating both patient and corresponding cost data. Hence, new research can be developed from our findings.

## Limitations

The study focused solely on direct costs; other non-medical expenses, such as transport to hospital, childcare, and work absenteeism, were not included, which could have led to an underestimation of the total estimated costs of UFs. Furthermore, the study was conducted in a hospital setting, whereas UFs are asymptomatic. Consequently, it is possible that we overlooked some cases when estimating the prevalence, which could have led to an underestimation of the total cost of UFs, especially the outpatient treatment and care costs. Another limitation was the absence of the index cost in Eswatini. We used the private price estimates for the best possible price estimates. Notwithstanding these limitations, we believe that our estimates present up-to-date evidence of the economic burden of UFs in the Kingdom of Eswatini.

## Conclusion

The study presents current estimates on the cost of uterine fibroids in the Kingdom of Eswatini. The findings indicated that the direct medical costs and economic burden of uterine fibroids are high, particularly given that the government pays for about 45% of all health care services and several other socioeconomic challenges faced by the country. This cost is likely to increase over time due to the high prevalence. As the cost of UFs significantly impacts patients, health systems, and the government of Eswatini, informing SRH policymakers and implementing cost-effective treatment alternatives will minimise the economic burden of UFs.

## Abbreviations

UFs: Uterine fibroids
COI: Cost of Illness
SRH: Sexual Reproductive Health
USD: United States Dollars
SZL: Swaziland Lilangeni
EHHRRB: Eswatini Health & Human Research Review Board
UKZN: University of KwaZulu-Natal
BREC: Biomedical Research Ethics
PPPs: Purchasing Power Parities
LMICs: Low- and Middle-Income Countries
OOP: Out of Pocket Payment
